# Identifying a confused cell identity for esophageal squamous cell carcinoma

**DOI:** 10.1038/s41392-022-00946-8

**Published:** 2022-04-13

**Authors:** Xiangyu Pan, Jian Wang, Linjie Guo, Feifei Na, Jiajia Du, Xuelan Chen, Ailing Zhong, Lei Zhao, Lu Zhang, Mengsha Zhang, Xudong Wan, Manli Wang, Hongyu Liu, Siqi Dai, Ping Tan, Jingyao Chen, Yu Liu, Bing Hu, Chong Chen

**Affiliations:** 1 Department of Gastroenterology, State Key Laboratory of Biotherapy and Cancer Center, West China Hospital, Sichuan University, Chengdu, Sichuan China; 2 Department of Thoracic Oncology, State Key Laboratory of Biotherapy and Cancer Center, West China Hospital, Sichuan University, Chengdu, 610041 Sichuan China; 3 Dpartment of Urology, Institute of Urology, West China Hospital, Sichuan University, Chengdu, China

**Keywords:** Head and neck cancer, Cancer genetics

## Abstract

The cell identity of malignant cells and how they acquire it are fundamental for our understanding of cancer. Here, we report that esophageal squamous cell carcinoma (ESCC) cells display molecular features equally similar but distinct to all three types of normal esophageal epithelial cells, which we term as confused cell identity (CCI). CCI is an independent prognostic marker associated with poor prognosis in ESCC. Further, we identify tropomyosin 4 (TPM4) as a critical CCI gene that promotes the aggressiveness of ESCC in vitro and in vivo. And TPM4 creates CCI through activating the Jak/STAT-SOX2 pathway. Thus, our study suggests an unrecognized feature of ESCC cells, which might be of value for clinic prognosis and potential interference.

## Introduction

The identity of malignant cells, distinguished from normal cells at various aspects, would be central for our understanding of this disease and provide insight for diagnosis and clinic interference. The hallmarks of cancer are the basic features of and fundamental principles underlying malignancies, which have been gradually revealed over the last half-century through extensive studies on the process of tumorigenesis and by comparing tumor cells with their normal counterparts.^1–3^

According to the traditional developmental view of tumorigenesis, it has long been proposed that tumorigenesis is an unusual event of normal development and malignant cells arise from the stem and/or progenitor cells with differentiation block.^4–11^ One of the best examples might be acute myeloid leukemia (AML), which has been suggested to be transformed from hematopoietic stem and progenitors with differentiation-blocking mutations.^6,10,12,13^ And thus, it is generally believed that cancer cells which are more like stem cells are more aggressive than those with differentiation features.^6,10,14,15^ However, there are remarkable exceptions, such as multiple myeloma, which is generally thought to be developed from mast cells, the terminally differentiated B lymphocytes.^16^ And recently, it has been argued that the so-called cancer stem cells and differentiated cells could two-way transit to each other, which, together with other arguments, challenges the developmental view of tumorigenesis.^17–19^

Recently, there is accumulating evidence suggesting that the lineage identity of cancer cells might be dynamic. For example, a subset of non-small cell lung cancer (SCLC) with estimated glomerular filtration rate mutations developed resistance of tyrosine kinase inhibitors and transformed into SCLC with RB1 loss.^20^ LKB1 mutations can promote the transit of lung adenocarcinoma into squamous cell carcinoma (SCC).^21^ And it has been proposed that a high-plasticity cell state might underlie lung adenocarcinoma with poor outcomes and chemoresistance.^22,23^ Similarly, p53 and Rb1 loss increase the lineage plasticity of antiandrogen resistant prostate cancer into neuroendocrine-like cancer.^24–26^ Thus, in the latest version, the unlocked phenotypic plasticity was added as a new hallmark of cancer.^2^

However, the cell identity of most types of cancers, especially those derived from epithelial cells, which consist of more than 90% of human malignancies, remains unclear.^27–29^ The molecular and cellular pathologies of SCC, one of the most common epithelial cancers with worse outcomes than others, are less understood. This type of cancer seems to be differentiated toward squamous epithelial cells to some extent and thus distinguishes it from epithelial stem cells. On the other hand, patients with low-grade differentiation have a poor prognosis.^11,29^ To resolve these paradoxes and gain a better understanding of the cellular identity and the underlying molecular mechanisms of SCC, we analyzed the cellular identity of esophageal SCC (ESCC) at single-cell resolution in the background of normal epithelial cells with a discovery cohort of seven tumor samples and one distal normal sample from five pathologically confirmed ESCC patients. We then validated the results in a cohort of 60 patients analyzed by single-cell RNA-seq (scRNA-seq) and 9 cohorts of a total of 534 patients analyzed by bulk RNA-seq or microarray assays.

## Results

### Single-cell transcriptomics reveals CCI as a unique identity of ESCC cells

**Fig. 1:**
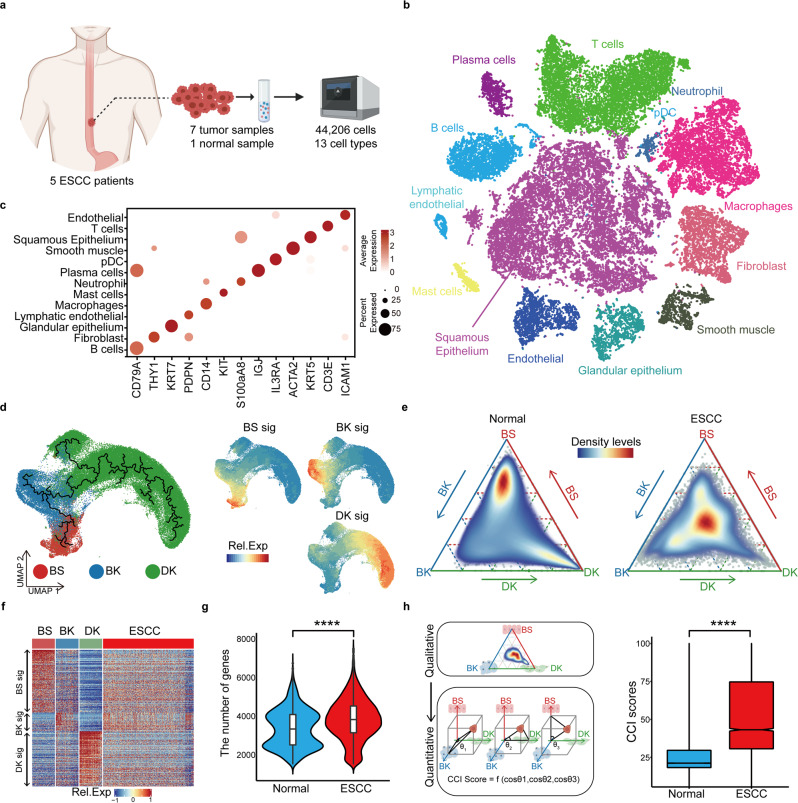
Confused cell identity (CCI) is a feature of ESCC cells at single-cell resolution. **a** The schematic graph of the experimental design. **b** The t-SNE map of single-cell ESCC landscape was colored by cell subtypes. **c** The dot plot showed the expression levels of classical signatures in each cell subtype. **d** The UMAP map of normal squamous epithelial (SE) was colored by cell subtypes. The black lines, fitted by monocle3, represented the development lineage of normal SE (left). The distribution of expression levels of BS, BK, and DK signatures on the UMAP map (right). **e** The ternary diagram showed the similarity/variation in scRNA-seq data of SE from the normal esophagus (left) and ESCC (right), with BS, BK, and DK cells. The color represented the density levels of distribution. **f** The heatmap showed the expression levels of BS, BK, and DK signatures in scRNA-seq data. **g** The boxplot showed the number of genes detected in normal and ESCC SE cells (*n* = 4052 cells, normal; *n* = 7580 cells, ESCC). *p* Values calculated by two-sided unpaired *t*-test. ****p < 0.0001. **h** The schematic graph of CCI scores calculation (left). The box plot showed the CCI scores in single-cell normal samples and ESCC samples (right, *n* = 4860 cells, normal; *n* = 6801 cells, ESCC). *p* Values calculated by Wilcoxon signed-rank test. ****p < 0.0001

The ESCC samples and normal esophageal tissue in the discovery cohort were analyzed with 10× Genomics scRNA-seq (Fig. 1a and Supplementary Fig. S1a, b). To better represent the squamous epithelial cells, we optimized the dissociation protocol to harvest more than a twofold number of epithelial cells than previous methods (Supplementary Fig. S1c).^30^ Total 44,206 cells were obtained, including 16,657 normal and malignant squamous epithelial cells. The squamous epithelial cells, glandular epithelial cells, immune cells, endothelial cells, and other tumor microenvironmental cells were separated and identified with classic markers (Fig. 1b, c, Supplementary Fig. S2a and Supplementary Table 1). On the t-SNE map, all these samples were essentially evenly distributed (Supplementary Fig. S2b–e).

All the esophageal squamous epithelial cells expressed high levels of marker gene KRT5 (Supplementary Fig. S2a). In patients, the squamous epithelial cells displayed obvious copy number variations, compared to other cells, calculated by inferCNV (Supplementary Fig. S2f). Thus, these cells were identified as ESCC cells. In normal esophageal tissues, the esophageal squamous epithelium consists of three major types of cells, including basal stem cells (BS), transient proliferating basal keratinocytes (BK), and postmitotic differentiated keratinocytes (DK).^31–33^ And these cells are distinct from each other by their morphologies, localization, and molecular features and BS can generate BK and then DK through stepwise differentiation.^32,33^ Consistently, we found that normal squamous epithelial cells could be divided into three continuous subpopulations (Supplementary Fig. S3a). Similar three subpopulations were observed with the combinations of 4885 cells from our dataset and the human cell atlas (the HCA) esophageal dataset (Fig. 1d). One of them expressed *SOX2*, *TP63*, and *KRT15*, suggesting them as BS, the second one expressed a high level of *KRT5* and the third one expressed markers *KRT14*, *KRT13*, and *IVL*, corresponding to the definition of BK and DK, respectively (Supplementary Fig. S3b, c and Supplementary Table 2). The unsupervised fate map showed a pseudo trajectory from BS to BK and DK at the end (Fig. 1d). BK had the highest proliferating score, while DK was mostly postmitotic (Supplementary Fig. S3d). And accordingly, BS had enrichments of stemness-related pathways, BK had enrichments of MYC targets, DNA replication, and other cell cycle pathways and DK had high levels of genes related to the functions of squamous epithelium, including cornification and keratinization (Supplementary Fig. S3e).

To visualize the relationship of ESCC cells with normal esophageal squamous epithelial cells, we created a ternary map with BS, BK, and DK at each corner (Fig. 1e). This map could also distinguish all normal esophageal squamous epithelial cells from other studies (Supplementary Fig. S4a, b).^34,35^ However, strikingly, all malignant cells fell at the center of the triangle with equal distance to each corner. This relative position of ESCC cells indicated that they might contain similar gene expressions as each of these three normal squamous epithelial cells. Indeed, the gene expression heatmap showed that ESCC cells expressed all the signature genes of BS, BK, and DK, though at slightly lower levels (Fig. 1f). And concordantly, the average numbers of genes detected in malignant cells were significantly more than those in normal cells (Fig. 1g). This result was in contrast to what the traditional stem cell view of tumorigenesis. We termed this new cell identity relative to the normal cells as the confused cell identity (CCI). To quantify the CCI of each cell on the ternary map, we designed a CCI score by calculating the standard deviation of the cosine values of the three angles with the dot of cell position as the vertex and the connecting lines of the dot to each corner and the origin as rays. The median CCI score was 21.2 for the normal cells and 43.2 for the malignant cells (Fig. 1h). With 44,730 malignant cells from 60 ESCC patients in a previous study, we also observed a similar CCI for these ESCC cells (Supplementary Fig. S4c).^30^ And although they were from independent studies and potential batch effects, the normal esophageal squamous cells had less than 20 CCI scores while ESCC cells had a score of 35.4 (Supplementary Fig. S4d, e). Thus, we proposed that CCI was a new molecular feature of malignant cells.

### CCI is a general feature of ESCC in multiple cohorts

**Fig. 2:**
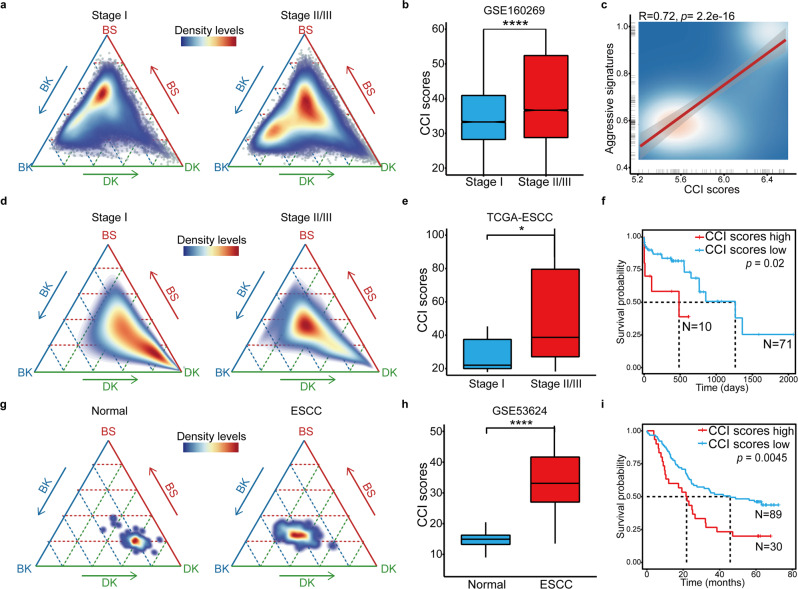
CCI is an independent prognostic marker in ESCC. **a** The ternary diagram showed the similarity/variation in stage I (left) and stage II/III (right) from scRNA-seq data of GSE160269, with BS, BK, and DK cells. The color represented the density levels of distribution. **b** The boxplot showed the CCI scores in stage I and stage II/III patients from GSE160269 (n = 13,041 cells, Stage I; *n* = 31,506 cells, Stage II/III). *p* Values calculated by Wilcoxon signed-rank test. ****p < 0.0001. **c** The scatter plot showed the correlation between the CCI scores and the expression levels of ESCC aggressive stage-specific signatures in scRNA-seq. **d** The ternary diagram showed the similarity/variation in stage I (left) and stage II/III (right) from bulk RNA-sea data of TCGA-ESCC, with BS, BK, and DK cells. The color represented the density levels of distribution. **e** The boxplot showed the CCI scores in stage I and stage II/III patients from TCGA-ESCC (*n* = 7 patients, Stage I; *n* = 72 patients, Stage II/III). *p* Values calculated by two-sided unpaired *t*-test. *p < 0.05. **f** The Kaplan–Meier survival curves of TCGA-ESCC patients with low and high CCI scores. **g** The ternary diagram showed the similarity/variation in adjacent normal (left) and ESCC (right) from array data of GSE53624, with BS, BK, and DK cells. The color represented the density levels of distribution. **h** The boxplot showed the CCI scores in adjacent normal and ESCC samples from GSE53624 (*n* = 119 patients, normal; *n* = 119 patients, ESCC). *p* Values calculated by Wilcoxon signed-rank test. ****p < 0.0001. **i** The Kaplan–Meier survival curves of GSE53624 patients with low and high CCI scores

Further, we wondered whether the CCI score was correlated with the aggressiveness of ESCC in patients. First, we found that the average CCI score was significantly higher in ESCC patients with aggressive progression (Fig. 2a). In Zhang’s cohort with 60 ESCC patients, there were 16 Stage I and 44 Stage II/III patients.^30^ Despite the inter- and intra-tumoral heterogeneity, the CCI scores of the early stage patients were significantly less than those of the advanced-stage patients (Fig. 2b). Consistently, at the single-cell level, the CCI scores were significantly positively correlated with the expressions of ESCC advanced stage-specific genes (Fig. 2c and Supplementary Table 3).

Given that most of the available gene expression data for ESCC patients were bulk transcriptomics analyzed with RNA-seq or microarray, we wanted to measure the CCI status of each patient with bulk transcriptomes. In the TCGA ESCC cohort with 81 patients, we found all stage II/III patients located in the center of our CCI map, while the stage I patients’ location was close to the corner of DK (Fig. 2d). The average CCI score of higher-grade patients was significantly higher than that of the stage I patients (22.1 vs. 38.7, *p* = 0.028) (Fig. 2e). More importantly, we found that patients with high CCI scores had significantly worse prognoses than those with low CCI scores (median survival time 484 vs. 1263 days, *p* = 0.02) (Fig. 2f). Further, the CCI analysis could also be applied with the microarray gene expression data. In Li’s cohort^36^ with 119 ESCC patients analyzed with microarray gene expression profiling, all the tumor samples were at the center of the CCI map while their adjacent normal tissue was close to DK (Fig. 2g). And their CCI scores were 33.1 and 14.9, respectively (Fig. 2h). Similarly, in this cohort, the survival of patients with high CCI scores was significantly shorter than that of those with low-CCI scores (21.83 vs. 45.83 months, *p* = 0.0045) (Fig. 2i). To test whether other clinic factors would affect the CCI score, we analyzed the correlations of multiple clinic parameters with the CCI score in the TCGA ESCC cohort. The results showed that there were no significant differences of sex, smoking, alcohol history, treatment, BMI, and other clinic factors between the CCI low and high patients (Supplementary Table 4). These results suggested that the CCI score could be an independent prognostic marker for ESCC patients.

### The molecular mechanism underlying CCI

**Fig. 3:**
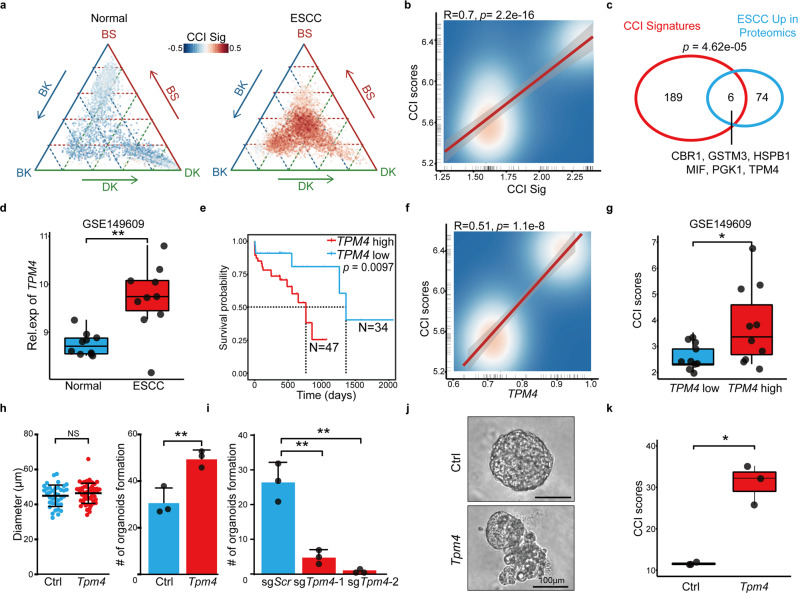
TPM4 is a CCI gene. **a** The ternary diagram showed the expression levels and distribution of CCI signatures in scRNA-seq from normal SE cells (left) and ESCC SE cells (right). **b** The scatter plot showed the correlation between the expression levels of CCI signatures and CCI scores in scRNA. **c** The Venn plot showed the intersection between CCI signatures and upregulated proteins in ESCC patients. The statistic power was calculated by a hypergeometric test. **d** The box plot showed the expression levels of *TPM4* in adjacent normal and ESCC samples from GSE149609 (*n* = 10 patients, normal; *n* = 10 patients, ESCC). *p* Values calculated by Wilcoxon signed-rank test. **p < 0.01. **e** The Kaplan–Meier survival curves of TCGA-ESCC patients with low and high expression of *TPM4*. **f** The scatter plot showed the correlation between the expression levels of *TPM4* and CCI scores in scRNA. **g** The box plot showed the log2-CCI scores in ESCC’s samples from GSE149609, which were grouped by the median of the expression levels of *TPM4* (*n* = 10 patients, *TPM4-*low; *n* = 10 patients, *TPM-*high). *p* Values calculated by two-sided unpaired *t*-test. *p < 0.05. **h** The dot plot showed the diameter of organoids with control and *Tpm4* overexpressed (left). The number of control and *Tpm4* overexpressed organoids in each well was counted (right). Data are shown as mean ± SD (*n* = 3 biological replicates, Ctrl; *n* = 3 biological replicates, *Tpm4*). *p* Values calculated by two-sided unpaired *t*-test. NS nonsignificant; **p < 0.01. **i** The number of sg*Scr* and sg*Tpm4* organoids in each well was counted. Data are shown as mean ± SD (*n* = 3 biological replicates, sg*Scr*; *n* = 3 biological replicates, sg*Tpm4-1*; *n* = 3 biological replicates, sg*Tpm4-2*). Three independent biological replicates were performed for each group. *p* Values calculated by two-sided unpaired *t*-test. **p < 0.01. **j** The morphology of control and *Tpm4* overexpressed esophageal organoid. Scale bar, 100 μm. **k** The boxplot showed the CCI scores in control and *Tpm4* overexpressed organoids (*n* = 3 organoids, Ctrl; *n* = 3 organoids, *Tpm4*). *p* Values calculated by two-sided unpaired *t*-test. *p < 0.05

Then we explored the molecular mechanism underlying CCI in ESCC. The CCI score was positively correlated with many cancer pathways, including those related to cancer stemness, metastasis, and epithelial cell proliferation (Supplementary Fig. S5a and Supplementary Table 5). Then, we identified the CCI signature genes by comparing the CCI-positive cells with the negative epithelial cells. Total 195 genes specifically expressed in the malignant cells were scored (Supplementary Fig. S5b and Supplementary Table 6); these CCI signature genes were also upregulated in the tumors of advanced stages compared to those of early stages (Supplementary Fig. S5b). On the CCI map, these CCI signature genes were not expressed in normal cells at the three corners and were highly expressed in the malignant cells at the center (Fig. 3a). Noteworthy, the expression levels of these CCI signature genes were significantly positively correlated with the CCI score (Fig. 3b).

To dissect the molecular mechanisms underlying CCI, we performed the network analysis on the CCI signature genes. The results showed that multiple gene networks were enriched for CCI, including spliceosome, ribosome, antigen processing and presentation, oxidative phosphorylation, and pathways in cancer (Supplementary Fig. S5c). Further, we looked for the CCI signature genes which were highly expressed in ESCC patients at the protein levels.^37^ Six of them, CBR1, GSTM3, HSPB1, MIF, PGK1, and TPM4 were identified (Fig. 3c). Among them was tropomyosin 4 (*TPM4*), which, by itself, had the highest diagnostic value in ESCC patients revealed by the multivariant Cox regression analysis (Supplementary Fig. S5d). *TPM4* was among the most upregulated genes in ESCC and various cancers at the protein level (Supplementary Table 7).^37–40^ In the GSE149609 cohort, we confirmed the upregulation of *TPM4* in ESCC patients (Fig. 3d). *TPM4* high expressions were significantly associated with poor prognosis (Fig. 3e). And the expression level of *TPM4* was significantly positively correlated with the CCI score at the single-cell levels and the patient level (Fig. 3f, g). Thus, we believed that *TPM4* was a CCI gene.

### TPM4 overexpression increases the CCI score

To test the function of *TPM4*, we overexpressed it in esophageal organoids. *TPM4* overexpression did not significantly affect the size of organoids. However, once passaged, the *TPM4* organoids gave rise to substantially more secondary organoids, indicating that *TPM4* promoted the self-renewal of progenitor cells (Fig. 3h). And reversely, disruption of *Tpm4* significantly reduced the formation of esophageal organoids (Fig. 3i). Thus, TPM4 might not be required for cell proliferation or growth but is essential for the self-renew of esophageal epithelial organoids. And interestingly, more than half of the *TPM4* overexpressed organoids displayed a dramatic morphology change those cells migrated out of the organoids (Fig. 3j and Supplementary Fig. S5e). *TPM4* can affect cell migration by regulating the formation of F-actin.^41,42^ And consistent, the migrating cells in the *Tpm4* overexpressed organoids expressed high levels of F-actin (Supplementary Fig. S5f).

In agreement with its function in the self-renewal and migration of esophageal epithelial cells, the *TPM4* expression was positively correlated with multiple related pathways, including EMT, stem cell maintenance, tumor invasiveness (Supplementary Fig. S6a). And the enriched pathways in the *TPM4* high ESCC samples were primarily overlapped with those in the CCI gene signature (Supplementary Fig. S5a). Then, we measured the CCI status of control and *Tpm4*-overexpressed organoids in the CCI map. While the control organoids were close to the corner of BK, those with *Tpm4* overexpression moved toward the center (Supplementary Fig. S6b and Supplementary Table 8). And the CCI scores of *Tpm4* overexpressed organoids were significantly higher than those of the control ones (Fig. 3k). Taken together, *TPM4* promoted CCI in esophageal epithelial cells.

### TPM4 promotes ESCC metastasis in mice

**Fig. 4:**
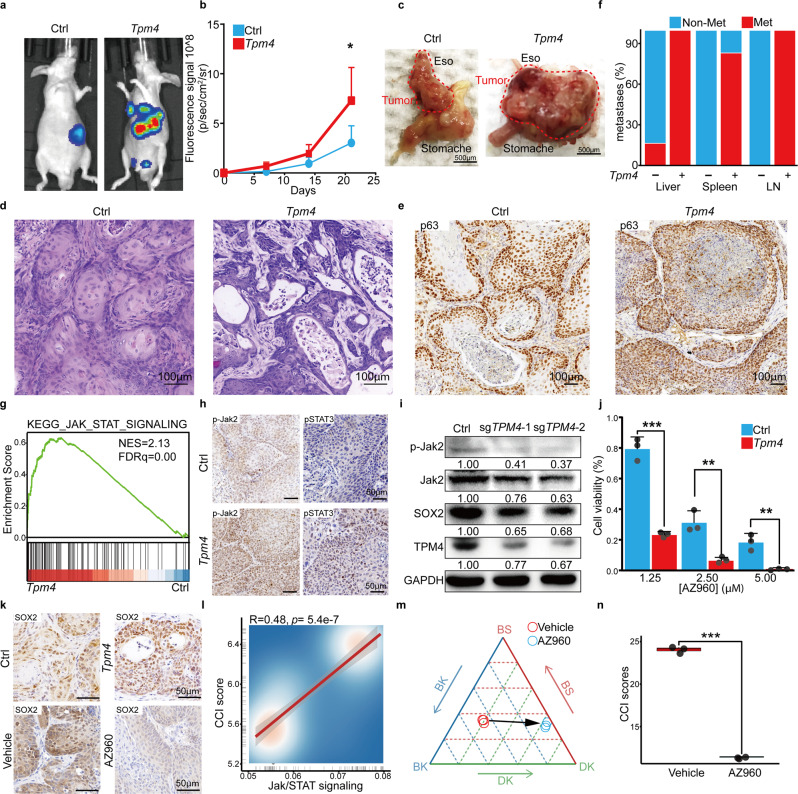
TPM4 promotes CCI and ESCC aggressiveness through the Jak/STAT pathway. **a** Representative living images after orthotopic transplantation of control (left) and *Tpm4* (right) overexpressed tumor organoids, each group consisting of six mice. **b** The luciferase fluorescence signal intensity of the control and *Tpm4* overexpressed mice after orthotopic transplantation. Data shows the means ± SEM. (*n* = 6 independent biological replicates were performed for each group). *p* Values calculated by two-sided unpaired *t*-test. *, *p* < 0.05. **c** Representative bright-field images of the orthotopic esophageal tumor tissue derived from the control (left) and *Tpm4* overexpressed (right). Scale bar, 500 μm. **d** Representative H&E staining images of primary orthotopic tumors derived from the control (left) and *Tpm4* overexpressed (right). Scale bar, 100 μm. **e** Representative immunohistochemistry images of p63 in orthotopic control and *Tpm4* overexpressed tumors. Scale bar, 100 μm. f The percentages of the control or *Tpm4* overexpressed mice with indicated numbers of metastases in liver, spleen, and lymph nodes (LN) were summarized. (Each group consists of *n* = 6 mice). **g** The GSEA plot showed the Jak/STAT signaling pathways enriched in *Tpm4* overexpressed samples. **h** Representative immunohistochemistry images of p-Jak2 and pSTAT3 in subcutaneous control and *Tpm4* tumors. Scale bar, 50 μm. **i** The representative western blotting pictures showed the p-Jak2, Jak2, SOX2, TPM4, and GAPDH levels in the control or *TPM4* disrupted organoids. **j** The viability of control and *Tpm4* overexpressed tumor organoids in different concentrations of AZ960. Data are shown as mean ± SD. (*n* = 3 independent biological replicates were performed for each group). *p* Values calculated by two-sided unpaired *t*-test. **p < 0.01; ***p < 0.001. **k** Representative immunohistochemistry images of SOX2 in subcutaneous control and *Tpm4* overexpressed tumors (top) and in subcutaneous *Tpm4* overexpressed tumors treated with vehicle and AZ960 (bottom). Scale bar, 50 μm. **l** The scatter plot showed the correlation between the expression levels of Jak/STAT and CCI scores in scRNA. **m** The ternary diagram showed the similarity/variation in RNA-seq data of *Tpm4* overexpressed organoids treated with vehicle and AZ960, with BS, BK, and DK cells. Three independent biological replicates were performed for each group. **n** The boxplot showed the CCI scores in *Tpm4* overexpressed organoids treated with vehicle and AZ960 (*n* = 3 organoids, Vehicle; *n* = 3 organoids, AZ960). Three independent biological replicates were performed for each group. *p* Values calculated by two-sided unpaired *t*-test. ***p < 0.001

We further confirmed the function of *TPM4* in ESCC in vivo. *Tpm4* was overexpressed in mouse ESCC organoids and then subcutaneously transplanted into recipient mice with control ESCC organoids on the other side. Tumors with *Tpm4* overexpression grew significantly faster than control tumors, measured by fluorescence living imaging (Supplementary Fig. S7a, b). Histology analyses suggested that *Tpm4* tumors were a more advanced stage than control ESCC, indicating a higher nuclear-to-cytoplasmic ratio, denser nuclei, and less differentiation (Supplementary Fig. S7c). And there were also significantly more Ki67+ tumor cells in *Tpm4* overexpression ESCC than control tumors (Supplementary Fig. S7d). Then, we orthotopically implanted the control and *Tpm4*-overexpressed ESCC organoids under the epithelial layer of the recipient’s esophagus. Fluorescence living imaging showed the *Tpm4* overexpressed tumors had a dramatically more vigorous fluorescence intensity than the control tumor (Fig. 4a, b). Consistently, the biopsy revealed that the massive tumors with *Tpm4* overexpression spread to the whole stomach while the control tumors were limited to the transplanted site (Fig. 4c). H/E and IHC staining confirmed that both tumors with and without *Tpm4* overexpression were typical ESCC expressing diagnostic markers p63 and CK14, highly similar to the human disease (Fig. 4d, e and Supplementary Fig. S7e). These pathologic analyses and p63 staining suggested that *Tpm4* overexpressed tumors were more aggressive than the control.

And intriguingly, we noticed strong fluorescence signaling at multiple sites on the recipient mice of *Tpm4* tumors (Fig. 4a). Biopsy showed that the *Tpm4* mice displayed specific signaling on numerous organs, including liver, spleen, and lymph nodes, hotspots of ESCC metastases in patients (Supplementary Fig. S7f). The histology analyses of metastases in lymph nodes and liver confirmed these tumor cells as ESCC metastases (Supplementary Fig. S7g). All six *Tpm4* recipients had metastases in the liver and lymph nodes and five of them had metastases in the spleen. In comparison, only one of the six recipients with control ESCC had metastases in the liver and none of them had metastases in other organs (Fig. 4f).

### The TPM4-Jak/STAT-SOX2 axis for CCI in ESCC

To gain insight into the TPM4-promoting CCI, we performed transcriptome analyses of control and *Tpm4* overexpressed esophageal organoids. The results showed that the Jak/STAT signaling pathway was among the most upregulated pathways by *Tpm4* (Fig. 4g, Supplementary Fig. S8a and Supplementary Table 9). And the advanced ESCC also expressed higher levels of these genes than early ESCC (Supplementary Fig. S8b). Of note, the IHC staining confirmed that *Tpm4* tumors had increased p-Jak2 and p-STAT3 levels compared to the control tumors (Fig. 4h). And similarly, western blotting showed that *Tpm4* overexpression increased the p-Jak2 level and reversely, *Tpm4* loss decreased the p-Jak2 level (Fig. 4i and Supplementary Fig. S8c). And importantly, ESCC organoids with *Tpm4* overexpression were significantly more sensitive to the Jak2 specific inhibitor AZ960 than the control ESCC organoids (Fig. 4j). AZ960 treatment also reduced the growth of *Tpm4* overexpressed ESCC in vivo, indicated by reduced Ki67 staining (Supplementary Fig. S8d).

It has been reported that the Jak/STAT pathway can upregulate *SOX2*, a key transcription factor for cell identity and ESCC progress.^24,25,43,44^ Indeed, ESCC tumors with *Tpm4* overexpression had increased SOX2 staining compared to control tumors (Fig. 4k). And consistently, western blotting showed that *TPM4* overexpression upregulated SOX2 expression while *TPM4* disruption downregulated it (Fig. 4i). Further, AZ960 treatment reduced the SOX2 level in vitro and in vivo (Fig. 4k and Supplementary Fig. S8e).

In the end, we found that the expression levels of the Jak/STAT signaling pathway genes were significantly positively correlated with the CCI score in ESCC (Fig. 4l). And importantly, inhibition of the Jak/STAT pathway by AZ960 significantly reduced the CCI scores of ESCC cells and drove the *Tpm4* overexpressed ESCC cells from the center to the DK side on the CCI map (Fig. 4m, n and Supplementary Table 10).

## Discussion

By comparing the single-cell transcriptomes of ESCC cells and their counterparts, we propose that CCI, a status with a mixture of molecular features of multiple types of normal squamous cells, is a hallmark of ESCC cells at the single-cell and patient level. The identification of CCI suggests that ESCC carcinogenesis is not a simple differentiation block of normal development, and instead, the tumor cells acquire a new identity with partial similarities to multiple normal cell types. CCI is a cell identity simultaneously possessing features of multiple cell types and thus distinguished from lineage plasticity that cells switch their identities from one lineage to other lineages.^26^ Given the similarity of ESCC cells to BS, BK, and DK, it might be possible that multiple types of squamous epithelial cells could be the cell of origin of this disease. Thus, CCI describes a previously unrecognized feature of malignant cells, which is distinguished from the developmental view of tumorigenesis or the lineage plasticity theory.^4–11,24–26^

We show that CCI is not only associated with the aggressiveness of ESCC cells but also a potential independent prognostic marker for patients. Since CCI is measured by a set of signature genes, which might be more stable than single genes. It is robust and can also apply to various transcriptome profiling methods, including microarray profiling, bulk RNA-seq, and scRNA-seq. It would be interesting to test it in further clinic practice for ESCC patients. Further, once the lineage identification of normal cell types has been established for other tissues, similar strategies might be used to test whether CCI also plays role in other human cancers and in other contexts, including drug resistance.

Multiple molecular pathways might be underlying CCI, such as spliceosome, ribosome, antigen processing and presentation, and oxidative phosphorylation. We found that *TPM4*, one of the top CCI signature genes which have not been functionally studied in ESCC, promotes the CCI and ESCC aggressiveness. Mechanistically, *TPM4* can activate the Jak/STAT signaling pathway, which further activates *SOX2. SOX2* is frequently amplified and/or upregulated in ESCC and recently it has been shown to be able to induce ESCC in mice.^45–49^ The Jak/STAT signaling pathway has been shown to be activated in various human cancers and serve as a clinic therapeutic target at least in some hematopoietic malignancies.^50^ It can coordinate stem cell proliferation and lineage differentiation.^51^ *SOX2* is also reported to be critical for lineage identification in both normal and tumor cells.^24–26,52^ Interestingly, the Jak/STAT signaling pathway itself is on the CCI gene network. Thus, we believe that these CCI signature genes might be functionally linked in ESCC cells, and their interactions together contribute to the unique identity of malignant cells.

Identifying CCI and understanding its underlying molecular mechanisms reveal the therapeutical vulnerability of ESCC. We show that inhibiting the Jak/STAT signaling pathway can decrease the CCI score of ESCC and reduce the growth of ESCC cells. Further studies on the potential benefits of the Jak/STAT inhibitors, some of which have been used for patients with other syndromes, for ESCC patients.

## Materials and methods

This study complied with all relevant ethical regulations and was approved by the Ethics Committee on Biomedical Research, West China Hospital of Sichuan University.

### Study subjects and biospecimens

Individuals with ESCC (*n* = 5) were recruited from West China School of Medicine/West China Hospital of Sichuan University (WCSM/WCH; Sichuan, China) between 2018 and 2019. None of the subjects underwent radiotherapy, chemotherapy, or immunotherapy prior to surgery. ESCC tumor tissue and normal adjacent tissue (≧5 cm from the tumor margin) were obtained after Lugol’s iodine solution staining. The pathological diagnosis was made by pathologists in the Department of Pathology, West China Hospital, Sichuan University, who confirmed that there were no tumor cells in normal tissues and no necrosis in tumor tissues, and the proportion of tumor cells in tumor tissues was more than 60%. Written informed consent was obtained from every subject and this study was approved by the Institutional Review Board of WCSM/WCH.

### Human esophageal squamous carcinoma cell lines

TE10 (#CL-0453) were purchased from Procell Corp and cultured in RPMI-1640 medium with 10% FBS supplemented, 10% heat-inactivated FBS (Gibco, #10099-141) and 1% penicillin–streptomycin (Gibco, #10378016). All cell lines were tested negative for mycoplasma. TE10 cell lines were infected with the SpCas9 lentiviral vector with sg*TPM4* or lentiviral containing *TPM4* cDNA. To identify the phosphorylated JAK2 and phosphorylated STAT3, all cell lines were stimulated with human IFN-γ (50 ng/ml, novoprotein, #CO14) for at least 48 h.

### Mice

All mice used in this study were maintained in the SPF level mouse facility at State Key Laboratory of Biotherapy of Sichuan University. All the animal experiment protocols have been approved by the institutional Animal Care and Use Committees of Sichuan University. *Rosa26-CAG-Cas9-iRES-GFP* mice hereafter referred to as Cas9 (Cat# JAX:026179, RRID: IMSR_JAX:026179), *Trp53*^−*/*−^ mice were obtained from The Jackson Laboratory (Cat# JAX:000664, RRID: IMSR_JAX:000664). Recipient nude mice were purchased from Hfkbio (Cat# BALB/cA-nu Mice). All animal experiments were performed in accordance with relevant guidelines and regulations and were approved by the West China Hospital, Sichuan University. Mice were deeply anesthetized by gas inhalation isoflurane before surgery or euthanasia.

### Murine esophageal organoids culture

Six to eight weeks old male mouse was sacrificed for culture. Esophagus tissue was separated and rinsed with cold phosphate-buffered saline buffer (Gibco, Cat# 10010002) supplemented with antibiotics (Gibco, Cat# 15240096) with vigorous shaking. After several times washing transferred the tissue to a new 15 ml centrifuge tube and minced with fine scissors. Then 5 ml 0.25% trypsin–EDTA (Gibco, Cat# 25200072) was added to digest the tissues in a 37 °C water bath for 1 h. When most of the tissue dissolved, the digestion reaction was stopped by a serum containing DMEM medium. Filtered the mixture through a 70-μm cell strainer (JET BIOFIL, #CSS-013-070) to obtain a single-cell suspension, then centrifuged at 400*g*, 5 min and washed once with phosphate-buffered saline (PBS) subsequently. After the liquid in the centrifuge tube was removed, the Matrigel (BD, Cat# 354230) was used to resuspend the cell pellet. After being mixed on ice evenly, 30 μl of the mixture was added to the bottom of the 48-well plates to form a hemispherical shape. After solidification, organoid medium was applied (DMEM/F12 (Gibco, Cat# A4192001) medium supplemented with GlutaMax (Gibco, Cat# 35050079), N2 Supplement (Gibco, Cat# 17502001), B27 Supplement (Gibco, Cat# 17502001), 1 mM N-acetylcysteine (Sigma, Cat# A0737), 10 mM Nicotinamide (Sigma, Cat# N0636), 50 ng/mL human epidermal growth factor (PEPROTECH, Cat# 900-M05), and 100 ng /mL fibroblast growth factor-10 (PEPROTECH, Cat# 400-29A), 10 μm Y27632 (Sellect, Cat# S6390), 500 nM A83-01 (Sellect, Cat# S7692), 10% conditioned medium containing Wnt3a (homemade), Noggin and R-spondin (homemade). After 3–4 days, the esophageal organoids would be seen. For passage, organoids were dissociated by TrypLE^TM^ Express (Gibco, Cat# 12605010) at 37 °C for 10 min. The process disrupted the spherical organoids into cell aggregates or single cell that were then embedded in fresh Matrigel.

### Genomic DNA extraction, PCR amplification

Five-hundred microlitre DNA lysis buffer (homemade, 10 mM Tris, 100 mM NaCl, 10 mM EDTA, 0.5% SDS 0.4 mg/ml proteinase K) was used to lysis cells at 55 °C for at least 2 h. The supernatant was combined with 500μL isopropanol, mixed, and centrifuged at 13,000 rpm for 10 min. Removed the supernatant and then washed with 1 mL 75% ethanol once. Volatilized at 55 °C for about 5 min, the DNA pellet was then dissolved in double-distilled water. One-hundred nanogram DNA was used for each genotyping PCR reaction. PCR reactions were performed on a ProFlex^TM^ PCR system (Applied Biosystems).

### Plasmid construction and virus production

The full-length *TPM4* and *Tpm4* cDNA were cloned into the LentiCRISPR-v2 puro (RRID: Addgene_52961) lentiviral vector, the full-length *Kras*^*G12D*^and *cMyc* cDNA was cloned into the MSCV- cDNA -IRES-luciferase retroviral vector, and the guide RNAs were cloned into the vectors as previously described. All plasmids’ sequences were confirmed by Sanger sequencing. Subsequently, these resulting plasmids were transfected with the helper plasmids psPAX2 (RRID: Addgene_12260), pMD2.G (RRID: Addgene_12259) into HEK293T (CCLV Cat# CCLV-RIE 1018, RRID: CVCL_0063) cells by the calcium phosphate transfection method. The virus in the supernatant at 36 and 48 h were respectively collected. And all the sequences of sgRNA used in this study were listed in Supplementary Table 11.

### CRISPR–Cas9 knockout organoids

Organoids were disassociated with TrypLE^TM^ for 15–30 min at 37 °C in a 15 ml conical tube. Pipetted several times every 5 min until most of them became single cells and then centrifuged at 400*g* for 5 min. The cells were resuspended with lentiviral supernatant and centrifuged for 1 h at 800*g* followed by another 2 h incubating at 37 °C. After that, cells were harvested and seeded with Matrigel in a 48-well plate. Two days later, 1 μg ml^−^^1^ puromycin (Gibco, #A1113803) was added to select the positive cells for several passages. To validate the efficiency of CRISPR in purified clones, their genome was extracted for PCR reaction and then T7E1 enzymes were performed. Sanger sequencing was used to validate the mutation status finally.

### Subcutaneous tumor growth assay

After one week of cells/organoids transplantation, the length and width of the tumor were measured using calipers every 3 days. Tumor size was calculated with the formula like this: (length × width^2^) × 0.5. For AZ960 treatment, after transplantation 1 week, mice were injected intraperitoneally with AZ960 (Sichuan Shuyan Ltd., #905586-69-8) at a dose of 40 mg/kg or vehicle daily.

### Living image assay

After transplanted subcutaneously or orthotopically with organoids, mice would be periodically imaged to detect the luciferase fluorescence signal intensity with IVIS Spectrum (PerkinElmer) system. The mice were anesthetized with isoflurane after 250 μl 15 mg/ml d-luciferin, Sodium salt (Bio Vision, #7903-1G) in PBS intraperitoneally injected.

### Subcutaneous and orthotopic implantation

For the organoid mouse model, the tumor-forming background we selected was the mutation of *Pten*, *Smad4*, and overexpression of *Myc* and *Kras*^*G12D*^ in the esophageal organs of *Trp53*^−/−^; Cas9 mice (TPSCK). The organoid model of this genotype could form ESCC at four weeks in immunodeficient mice. The H&E and IHC staining had confirmed the pathological characteristic of ESCC generated from TPSCK organoids. Of note, organoids should be dissociated to approximately single cells using TrypLE^TM^ mentioned above before subcutaneous and orthotopic transplantation. For the AZ960 treatment experiment, one well of TPSCK/ TPSCK *Tpm4* tumor organoids were injected subcutaneously into the flank of nude mice (6–8 weeks old, male, six mice per group). For tumor formation of orthotopic implantation from primary TPSCK/TPSCK *Tpm4* overexpressed organoids, 2 wells of organoids were collected with 30 μl of the mixture (1:1 Matrigel/medium ratio) for orthotopically injecting into the esophagus of nude mice (6–8 weeks old, male, six mice per group). The nude mice were anesthetized with isoflurane, and an incision was made through the left upper abdominal pararectal line and peritoneum. The stomach and esophagus were carefully exposed. With fixing the stomach by forceps, the cells were slowly injected into the junction of the esophagus and stomach with an insulin needle. The abdominal wall and skin were closed with 5-0 Ethicon sutures (ET-661G). The entire process was performed on a clean bench, and the wound was disinfected with iodophor after the operation.

### Immunoblotting

Cells were lysed in lysis buffer (50 mM Tris-HCl pH 7.4, 150 mM NaCl, 1% Triton-X100 supplemented with 1 mM phenylmethylsulfonyl fluoride before use). Cell lysate in SDS loading buffer (50 mM Tris-HCl (pH 6.8), 10% glycerol, 2% SDS, 1% 2-mercaptoethanol, 0.1% bromophenol blue) was boiled and analyzed by SDS-PAGE (Bio-Rad) and transferred to 0.45 μm polyvinylidene fluoride (PVDF) membranes with transfer buffer (25 mM Tris base, 190 mM glycine, 20% methanol, and 80% ddH_2_O, pH 8.3). Then the PDVF membranes were blocked with 2% milk (2% bovine serum albumin for phosphorylated antibody) with TBST buffer (150 mM NaCl, 20 mM Tris-HCl pH 7.6, and 0.1% Tween-20) for 1h before incubation with primary antibodies for 2 h at room temperature or 4 °C overnight. After washing with TBST, membranes were incubated with secondary antibody for 1 h and then washed three times with TBST. Cells need to be treated with 50 ng/mL IFN-γ 48 h in advance for phosphorylated protein detection. The antibodies, including anti-Jak2 (Cell Signaling Technology Cat# 3230, RRID: AB_2128522), anti-pJak2 (Cell Signaling Technology Cat# 3771, RRID: AB_330403), anti-STAT3 (Cell Signaling Technology Cat# 12640, RRID: AB_2629499), anti-pSTAT3 (Fluidigm Cat# 3158005, RRID: AB_2661827), anti-TPM4(Abcam Cat# 181085), anti-SOX2 (Abcam Cat# ab92494, RRID: AB_10585428), anti-GAPDH (BioXcell, #BX-008) were used in this study.

### H&E, immunohistochemistry, and immunofluorescence staining

Fresh tumor tissues were fixed in 4% PFA, embedded in paraffin, and cut into 5-μm -thick sections. The sections were stained with hematoxylin for 1 min to identify the cell nucleus and eosin for 30 s to identify the cytoplasm. For immunohistochemistry, the sections were incubated with antibodies of pJAK2 (Abcam Cat# ab32101, RRID: AB_775808), pSTAT3 (Abcam Cat# ab267373), Sox2(Abcam Cat# ab92494, RRID: AB_10585428) and Ki67 (Abcam Cat# ab16667, RRID: AB_302459) overnight at 4 °C after process of antigen retrieval and block with 2% goat serum. After being washed with PBST, the sections were incubated with secondary antibodies at room temperature for 1 hour. Then DAB was applied for signal detection (ZSGB-BIO, ZLI-9018), followed by staining with hematoxylin for seconds and rinsed with water for 1 min. Then the sections were blocked with the Permount mounting medium. For immunofluorescence staining, deparaffinization and rehydration procedures were as described above, antibodies of P63 (Abcam Cat# ab735, RRID: AB_305870), CK13 (Leica Biosystems Cat# NCL-CK13, RRID: AB_563792), CK14 (ARP American Research Products Cat# 03-GPCK14, RRID: AB_1541011) and CK5 (Thermo Fisher Scientific Cat# MA5-17057, RRID: AB_2538529) were used. After secondary antibodies were added, the slides were mounted by mounting solution with DAPI (Solarbio, Cat#C0060). And the images were taken with fluorescence microscopy (Zeiss, 880).

### Organoid growth assay

For organoid growth assay, 2000 single cells were cultured in a 96-well plate embedded in 10 μl Matrigel (3 independent biological replicates were contained in each group). And the number of organoids was counted after 5 days of culture.

### JAK2 inhibitor treatment assay

For drug response, 1000 single cells were cultured in a 96-well plate embedded in 10 μl Matrigel and then subjected to different concentrations of AZ960 (Sellect, #S2214) or DMSO. After treatment for 48 h, the number of organoids was counted in each well. The number of organoids in the experimental group normalized by the DMSO group was the cell viability.

### Statistical analysis

The unpaired Student’s *t*-test (two-tailed) was used to evaluate the gene expression, tumor volume, organoid number. Expression differences among three groups were analyzed by ANOVA. The statistical analysis was performed using GraphPad Prism (RRID: SCR_002798) version 9. Data are represented as mean ± SEM. All results are considered statistically significant at *P* < 0.05.

### Bulk RNA-seq data analysis

TRIzol reagent (Invitrogen, Cat# 15596018) was used to extract the total RNA in each sample. The library was constructed by Illumina Stranded mRNA Sample Preparation Kit (NEB, #E7770) followed by the manufacturer’s protocol. And the Illumina NovaSeq 6000 was used to sequence with paired-end 150 bp.

The RNA-seq data were aligned by STAR (v2.6) (RRID: SCR_004463) with genome reference mm10 for mice and hg19 for humans. The quantification of transcripts in each sample was implemented by R packages, GenomicAlignments. DESeq2 (RRID: SCR_000154) was used to identify the differentiated expressed genes with |log2-FC| > 1 and *p*-value < 0.05. The GO and KEGG (RRID: SCR_012773) enrichment was performed by clusterprofliler. The normalized data, generated by DESeq, was processed by log-transformation for subsequent visualization and quantification of specific signatures.

### Single-cell RNA-seq data analysis

The library was prepared by using the 10× genomics platform, Chromium Single Cell 3′ Reagent Kits v3, following the manufactory’s protocol. The average 7000 total cells (from 7000 to 9000) of each sample were added to the individual channel with an average recovery of 5525 cells. The Gel Beads were used to partition cells in the Chromium instrument. After library generating, Illumina NovaSeq 6000 was used to sequence with paired-end 150 bp for each sample.

The cellranger (v2) was used to align the clean reads with genome reference hg19. The Seurat (v3) pipeline was used to quantify and visualize single-cell RNA data. The poor-quality cells whose detectable gene number is lower than 200 or higher than 7,500, and whose mitochondrial genes expression proportion is higher than 20%, would be removed. The poor-quality genes that detected lower than 3 cells also would be removed.

The vst model was used to identify 4000 high variable genes for subsequent processing. To reduce the systematic and random batch effects between samples, the embedding of harmony was used to cluster the population and reduce dimension. And the 30 statistically significant PCA components were used for harmony embedding calculation. The t-SNE and UMAP maps were generated from the Seurat formula, RunTSNE, and RunUMAP, respectively. The phase maps were generated from phateR. Each subpopulation was identified by the classical signatures, and top markers could be available. The inferCNV was used to identify the malignant squamous epitheliums cells compared with immune cells and other epitheliums in an individual sample.

The monocle (v3) was used to inference the normal squamous epithelium development lineage. The principal graph of phate and UMAP map was learned by reversed graph embedding firstly. Then, the formula order_cells, assigned the pseudo-time value in each cell based on their projection. Besides, slingshot also was used to confirm the predicted tumor lineage with 300 approximate points.

### Gene-sets enrichment and gene signatures identified

All the enrichment gene-sets were downloaded from GSEA (v7 database). Gene-sets variation analysis (GSVA) in omics data was performed on the pseudo-bulk transformation data, which normalized single-cell data into 100 bins to accelerate the speed and save the memory of calculation. The proliferation scores of the epithelium subpopulation were calculated and quantified based on the GSVA scores from proliferation-related pathways. Besides, the cell cycle status in the normal single-cell esophageal squamous epitheliums cells was calculated and quantified by CellCycleScoring with g2m genes and s specific genes.^53,54^ The percentages of cell cycle status had been summarized and visualized by ggplot2.

The signatures of subpopulations were detected by FindAllMarkers with 0.25 thresholds and 0.25 minimum fractions. And the BS, BK, and DK signatures were identified with pct.2 < 0.4. The progression signatures of ESCC were identified from commonly upregulated in stage II/III patients compared with stage I patients in TCGA-ESCA^55^ and GSE160269.^30^ And the CCI signatures were identified by CCI SE exclusively upregulated genes with fold-change > 0.2, base-mean > 0.8, and pct.1 > 0.8 compared with normal SE.

### CCI scores quantification

To quantify the expression levels of specific pathways or gene-sets on transcriptome data, the normalized matrix was used to calculate, as following formula processing

$$SSi=\frac{{\sum }_{i=1}^{N}Mi}{\rm{Number}}\,{\rm{of}}\,{\rm{genes}}$$

*Mi* means gene value in sample/single-cell *i*, *N* means the number of pathways/gene-sets, and *SSi* means signature score in sample/single-cell *i*.

After BS, BK, and DK signatures were quantified in each sample/single cell, ggtern was used to quantify the distribution of them on BS, BK, and DK dimensions and project the distribution of dots/samples on the ternary maps. The two-dimension kernel density of distribution on the ternary map was estimated by stat_density_tern with geom = ‘polygon’ and 500 bins. To better compare the distribution on the ternary map in different conditions, the same measurement scale of three axes should be reserved in each sample/single cell. Hence, the limits were set to the same number in each sample/single cell. And all visualization was complemented by package ggtern and ggplot2.

To make the qualitative ternary map quantitative, the CCI scores were created to define the distribution of each sample/single cell among BS, BK, and DK dimensions. Firstly, we defined three angles *θ*1–*θ*3 to descript the variation among three axes with sample *S* (*a*, *b*, *c*), respectively. Each angle exists between the one axis and the segments included the origin point and sample *S* (*a*, *b*, *c*). Based on the regular cosine law, we could generate the cosine value of each angle, as the following formula:

$$\begin{array}{l}\cos \theta 1=\frac{{a}^{2}+{\sqrt[2]{{a}^{2}+{b}^{2}+{c}^{2}}}^{2}-{\sqrt[2]{{b}^{2}+{c}^{2}}}^{2}}{2\times a\times \,\sqrt[2]{{a}^{2}+{b}^{2}+{c}^{2}}}\\\qquad \quad=\frac{a}{\,\sqrt[2]{{a}^{2}+{b}^{2}+{c}^{2}}}\end{array}$$

$$\begin{array}{l}\cos \theta 2=\frac{{b}^{2}+{\sqrt[2]{{a}^{2}+{b}^{2}+{c}^{2}}}^{2}-{\sqrt[2]{{a}^{2}+{c}^{2}}}^{2}}{2\times b\times \,\sqrt[2]{{a}^{2}+{b}^{2}+{c}^{2}}}\\\qquad\quad=\frac{b}{\,\sqrt[2]{{a}^{2}+{b}^{2}+{c}^{2}}}\end{array}$$

$$\begin{array}{l}\cos \theta 3=\frac{{c}^{2}+{\sqrt[2]{{a}^{2}+{b}^{2}+{c}^{2}}}^{2}-{\sqrt[2]{{a}^{2}+{b}^{2}}}^{2}}{2\times c\times \,\sqrt[2]{{a}^{2}+{b}^{2}+{c}^{2}}}\\\qquad\quad=\frac{c}{\,\sqrt[2]{{a}^{2}+{b}^{2}+{c}^{2}}}\end{array}$$

The *a*, *b*, *c* represented the BS, BK, and DK signatures value of sample *S*. And the *θ*1, *θ*2, *θ*3 represented the angles generated from sample *S*.

Next, we calculate the inverse value of standard deviation among three cosine values, and after multiplying by a constant *f*, CCI scores would be generated in each sample/single cell.

$${\rm{CCI}}\,{\rm{Score}}=\frac{f}{sd(\cos \theta 1,\,\cos \theta 2,\,\cos \theta 3)}$$

### CCI signature identification

To better identify the CCI signature genes, we designed a workflow for analyze. Firstly, we analyzed the distribution of the CCI scores of ESCC cells. The area under the curve (AUC) of CCI scores distribution had been calculated. Of note, the AUC curve was significantly fitted to the GLM binomial distribution. And the first intersection point between the AUC curve and predicted curve would be the cut-point of CCI scores for ESCC cells on the single-cell ternary diagram for calculating the CCI signature genes. The ESCC cells with their CCI scores higher than the cut point were used for identifying the CCI signature genes. Then we calculated the CCI signature genes by comparing these CCI positive cells with other squamous epithelium cells with the standards of fold-change > 0.25, base-mean > 0.8, and pct.1 > 0.8. And 195 genes were identified as the CCI signature genes for ESCC (Supplementary Table 6).

### Omics data analysis of ESCC database

The array data of transcriptome were downloaded by GEOquery, including GSE20347,^56^ 17 paired normal and ESCC samples; GSE23400_1,^57^ 53 paired normal and ESCC samples; GSE23400_2,^58^ 51 paired normal and ESCC samples; GSE70409,^59^ 17 paired normal and ESCC samples; GSE53624,^36^ 119 paired normal and ESCC samples. The probe id in GSE53624 had been converted to gene name by GPL18109 platform, and others had done by feature data of getGEO results.

The TPM data of bulk RNA-seq and the data of proteomics from GSE149609^37^ had been used in this study. In TCGA-ESCA cohorts, the 81 ESCC patients were extracted to analyze. Besides, the transcriptome data, survival information, and clinical classification of ESCC patients, from TCGA-ESCC and GSE53624 cohorts, were used to validate the clinical correlation between CCI and ESCC diagnosis. The Cox regression and the Kaplan-Meier survival curves were calculated and visualized by R package, survminer and survival. The determination of the cut-points for numerical variables in survival plots was based on the maxstat package, which also was integrated into the survminer package.

The single-cell data of normal esophageal tissue was downloaded from HCA projects and the human cell landscape. And squamous epitheliums were extracted following the origin annotation. After the same scRNA data pipeline analysis, the HCA and our normal sample were combined to reduce the dimension, and the UMAP map of normal esophageal squamous epitheliums was generated, and the expression levels of classical genes and gene-sets were projected and summarized.

The single-cell data of ESCC were downloaded from GSE160269. The squamous epitheliums were identified following the origin annotation. The clinical information of ESCC patients was used to divide patients into two groups including stage I and stage II/III.

## Supplementary information

Supplementary Table

Supplementary Figures

## Data Availability

The raw data of RNA-seq and scRNA-seq data in this study are deposited in NCBI GEO (GSE188955).

## Materials availability

The processed files and codes can be found in GitHub.
