# Supplementary material for: Identifying a confused cell identity for esophageal squamous cell carcinoma
Source: Signal Transduct Target Ther. 2022 Apr 13;7:122. doi: 10.1038/s41392-022-00946-8 (PMC9008022; doi:10.1038/s41392-022-00946-8)
Supplement: Supplementary file 2 — Supplementary Figures [file 41392_2022_946_MOESM2_ESM.docx]

Supplementary Materials for

Identifying a confused cell identity for esophageal squamous cell carcinoma

Xiangyu Pan, Jian Wang, Linjie Guo, Feifei Na, Jiajia Du, Xuelan Chen, Ailing Zhong, Lei Zhao, Lu Zhang, Mengsha Zhang, Xudong Wan, Manli Wang, Hongyu Liu, Siqi Dai, Ping Tan, Jingyao Chen, Yu Liu*, Bing Hu*, Chong Chen*

Correspondence to: yuliuscu@scu.edu.cn (Y.L), hubingnj@163.com (B.H) and chongchen@scu.edu.cn (C.C)

**This PDF file includes:**

Supplementary Fig. S1 (Related to figure 1)

Supplementary Fig. S2 (Related to figure 1)

Supplementary Fig. S3 (Related to figure 1)

Supplementary Fig. S4 (Related to figure 2)

Supplementary Fig. S5 (Related to figure 3)

Supplementary Fig. S6 (Related to figure 3)

Supplementary Fig. S7 (Related to figure 4)

Supplementary Fig. S8 (Related to figure 4)

Supplementary Table 1. The top markers of subpopulation in the single-cell landscape of ESCC

Supplementary Table 2. The signatures of BS, BK and DK cells in single-cell normal esophageal SE cells

Supplementary Table 3. The ESCC aggressive stage-specific signatures were identified from GSE160269 and TCGA-ESCC.

Supplementary Table 4. The clinical parameters in the CCI low and CCI high ESCC patiens

Supplementary Table 5. The CCI enriched pathways.

Supplementary Table 6. The signatures of CCI.

Supplementary Table 7. The significantly up-regulated proteins in ESCC.

Supplementary Table 8. The counts and differentiated expressing genes in TPM4 overexpression compared with control samples.

Supplementary Table 9. The KEGG enrichment of TPM4 overexpression compared with control samples.

Supplementary Table 10. The counts and differentiated expressing genes in AZ960 treatment compared with vehicle treatment.

Supplementary Table 11. The sequence of guide RNA for CRISPR/cas9 knock out


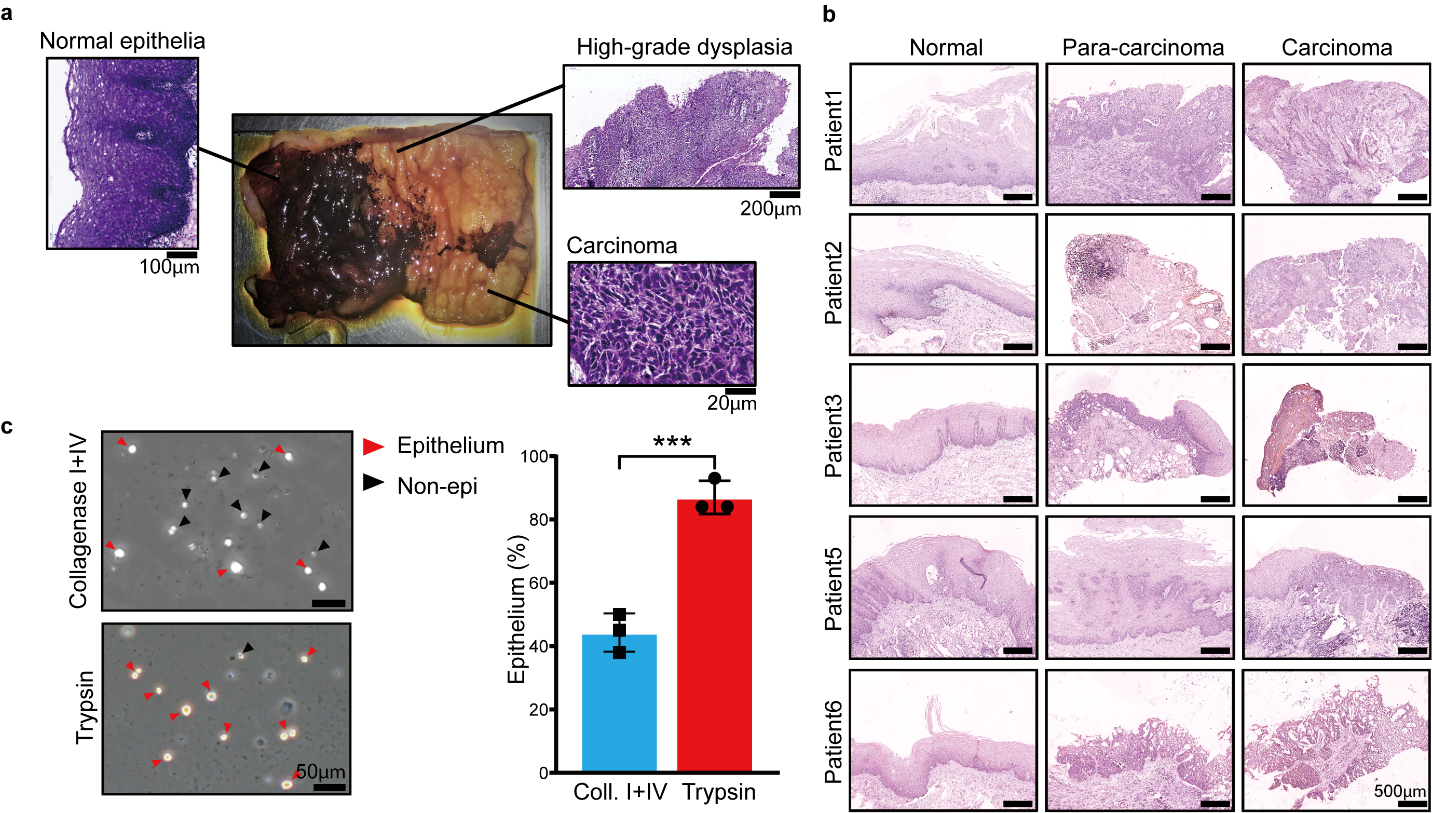


Supplementary Fig. S1. Pathologyof ESCC patients.

1. Representative H&E staining images of ESCC patients. Scale bar, 20μm, 100μm and 200μm.
2. Representative H&E staining images of each ESCC patient. Scale bar, 500μm.
3. The represented pictures of lysis tissue during the scRNA-seq data library construction (left). The bar plot summarized the number of epithelium cells lysis by collagenase I+IV and trypsin (right)(n=3 visual fields, Coll. I+IV; n=3 visual fields,Trypsin). *p* values calculated by two-sided unpaired t-test. ***, *p* < 0.001. Scale bar, 50μm,


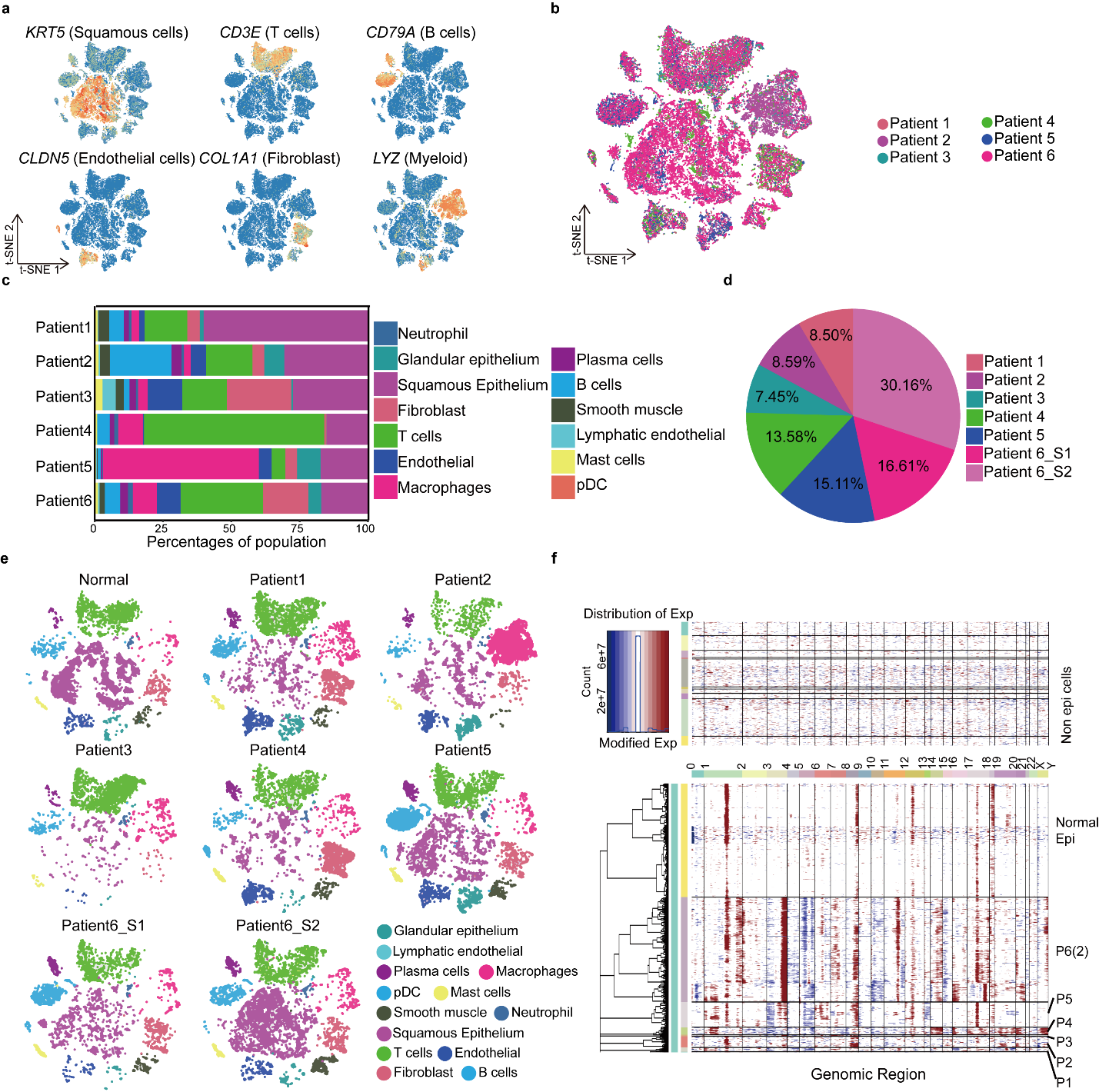


Supplementary Fig. S2. The single-cell landscape of ESCC.

1. The expression levels of *KRT5, CD3E, CD79A, CLDN5, COL1A1* and *LYZ* were projected on a single-cell ESCC landscape.
2. The t-SNE map of single-cell ESCC landscape was colored by patients.
3. The bar plot showed the percentages of each cell subtype among patients.
4. The pie charts showed the percentages of the squamous epithelium cells in each ESCC samples.
5. The t-SNE map showed the indivivual samples’ distrubtion of single-cell ESCC landscape, which colored by the cell population.
6. The inferCNV results of ESCC patients’ data.


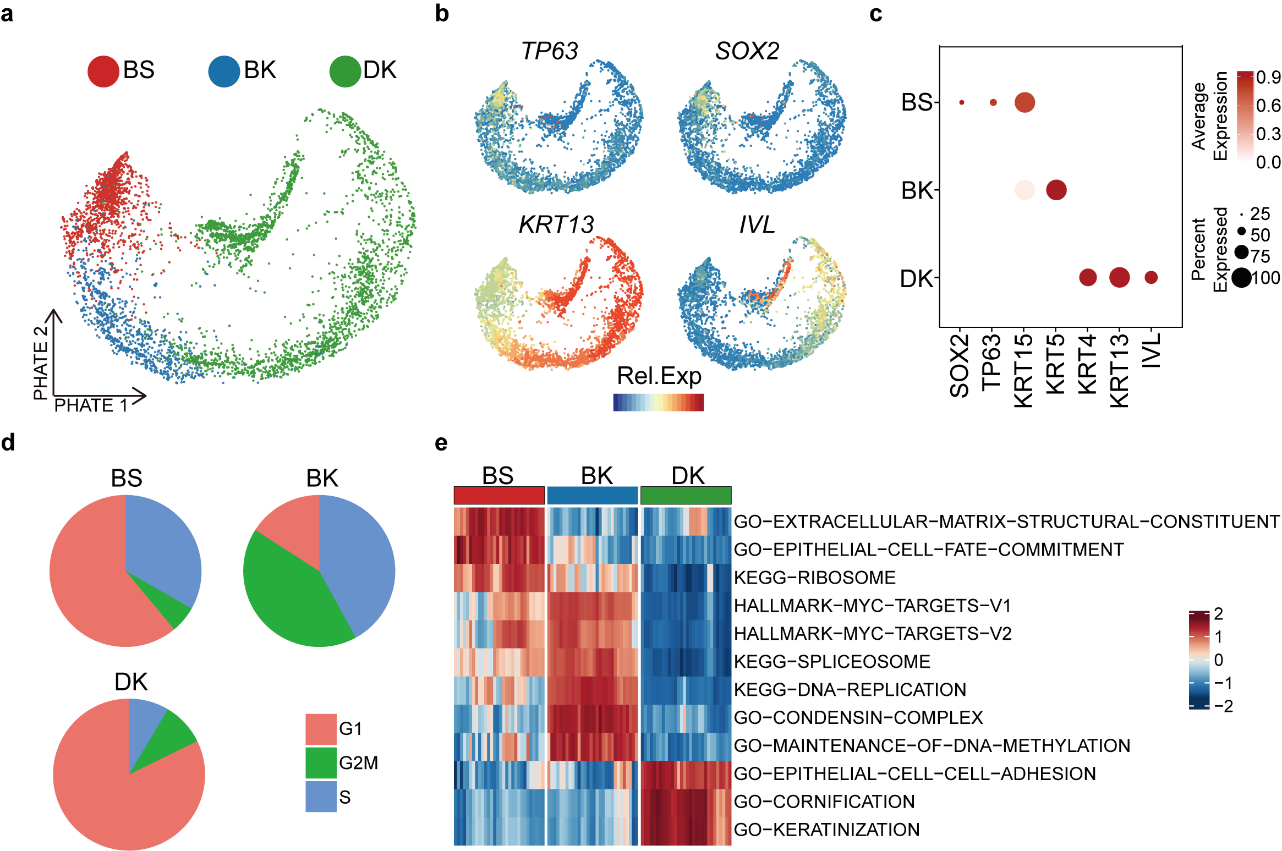


Supplementary Fig. S3. The single-cell landscape of normal esophageal squamous epitheliumns.

1. The PHATE map of normal esophageal squamous epitheliumn (SE) was colored by cell subtypes.
2. The expression levels of *TP63, SOX2, KRT13* and *IVL* were projected on the single-cell normal esophageal SE map.
3. The dot plot showed the expression levels of classical signatures in each SE subtype.
4. The pie charts summarized the phase of the cell cycle in each SE subtype.
5. The heatmap showed the GSVA results in each SE subtype.


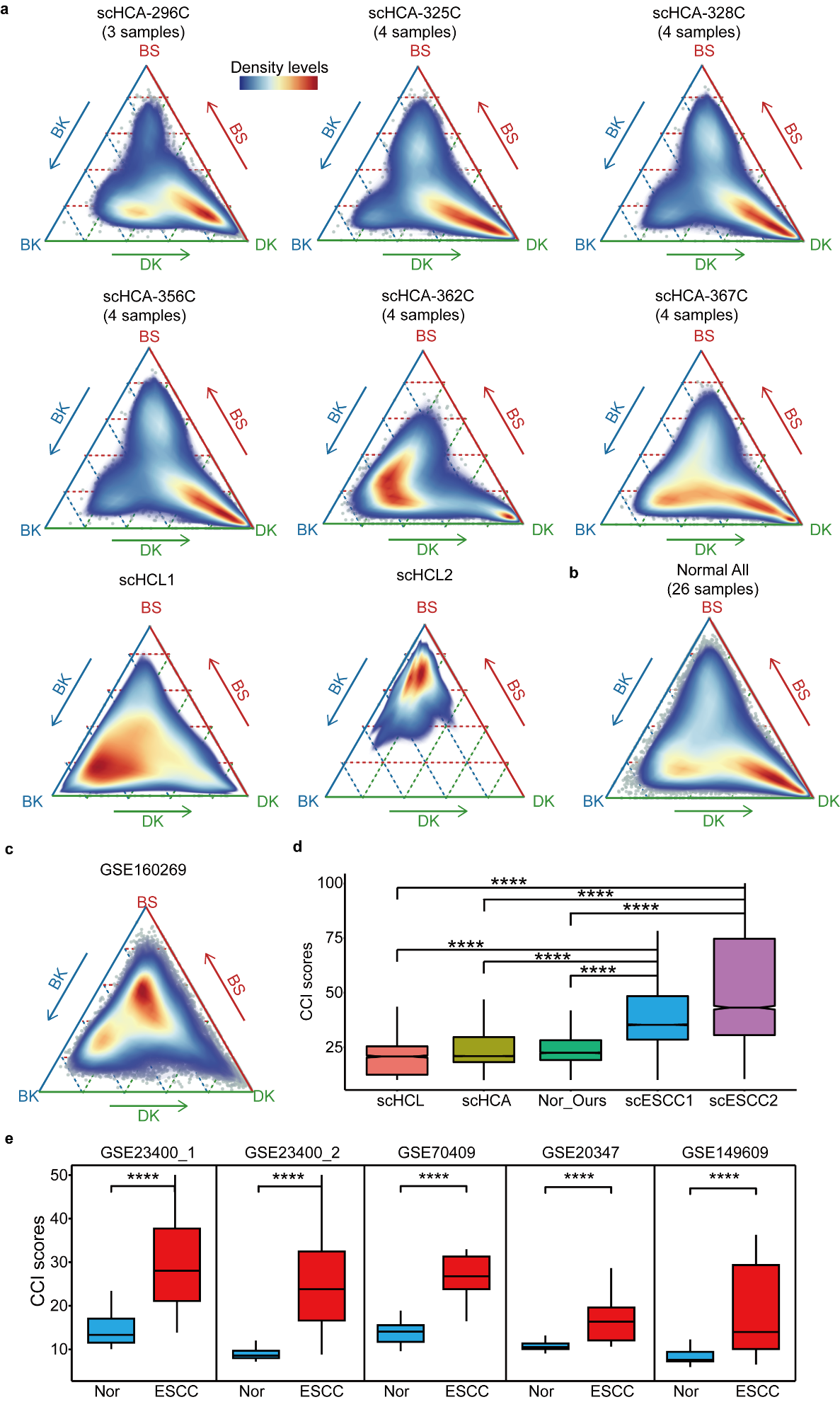


Supplementary Fig. S4. The CCI status of normal and malignant esophageal epithelial cells.

1. The ternary diagram showed the similarity/variation of SE in normal SE from scHCA and scHCL, with BS, BK and DK cells. The color represented the density levels of single-cell distribution.
2. The ternary diagram showed the similarity/variation of SE in normal SE from all samples, with BS, BK and DK cells. The color represented the density levels of single-cell distribution.
3. The ternary diagram showed the similarity/variation of ESCC SE from GSE160269, with BS, BK and DK cells. The color represented the density levels of single-cell distribution.
4. The boxplot showed the CCI scores in scHCL, scHCA, our normal scRNA data and ESCC scRNA data (n=1,740 cells, scHCL; n=80,320 cells, scHCA; n=4,885cells, Nor_Ours; n=44,547 cells, scESCC1; ; n=6,801 cells, scESCC2). *p* values calculated by Wilcoxon signed-rank test. ****, *p* < 0.0001.
5. The boxplot showed the CCI scores in adjacent normal samples and ESCC samples from miuitiple datasets (n=53 patients, Nor; n=53 patients, ESCC; n=51 patients, Nor; n=51 patients, ESCC; n=17 patients, Nor; n=17 patients, ESCC; n=17 patients, Nor; n=17 patients, ESCC; n=10 patients, Nor; n=10 patients, ESCC). *p* values calculated by Wilcoxon signed-rank test. ****, *p* < 0.0001.


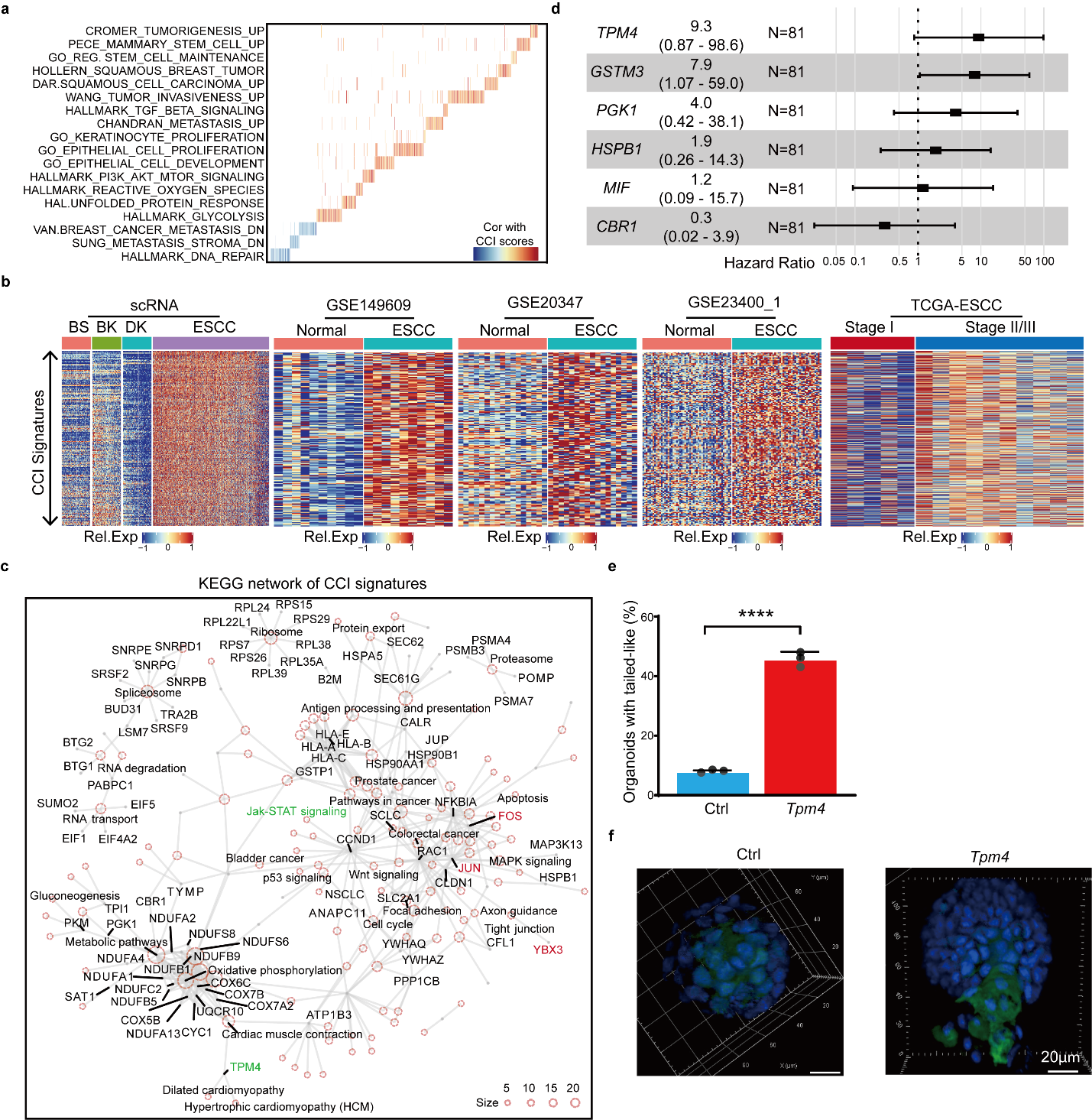


Supplementary Fig. S5. Identifying *TPM4* as a CCI gene.

1. The heatmap showed the GSEA results based on the correlation coefficient with CCI scores in ESCC squamous epitheliumn cells.
2. The heatmap showed the expression levels of CCI signatures in scRNA-seq, GSE149609 and GSE20347, GSE23400_1 and TCGA-ESCC.
3. The KEGG network of CCI signatures from single-cell data.
4. Multivariate Cox regression analysis of diagnostic CCI signatures.
5. The percentages of the organoids with tail-like structures in each well were counted. Data are shown as mean ± SD. (n=3 biological replicates, Ctrl; n=3 biological replicates, *Tpm4*). *P* values calculated by two-sided unpaired t-test. ****, *p* < 0.0001.
6. Representative immunofluorescence images in control (left) and *TPM4* overexpressed (right) organoid with Phalloidin (green), and 4′,6-diamidino-2-phenylindole (DAPI) (blue), Scale bar, 20 μm.


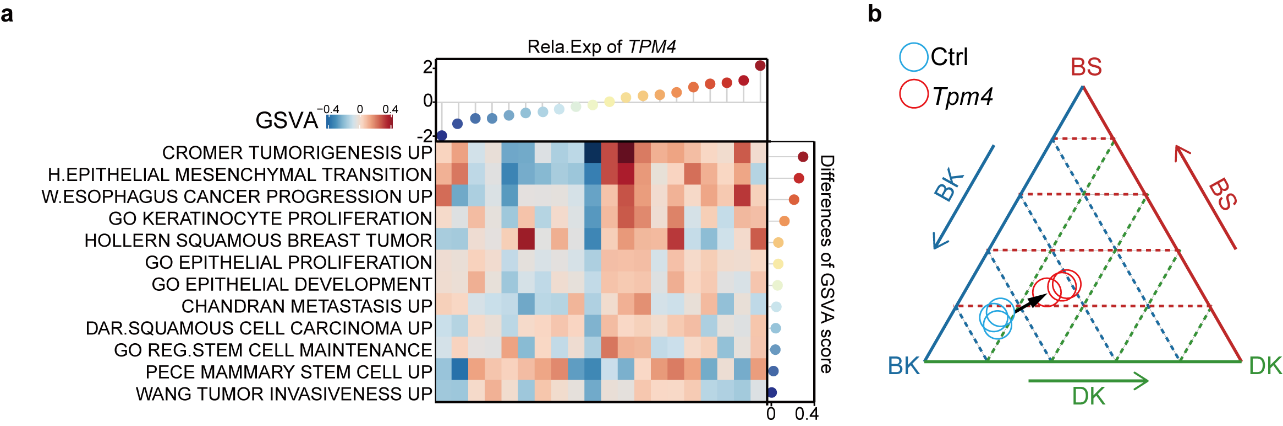


Supplementary Fig. S6. *TPM4* enhances CCI in esophageal epithelial cells.

1. The heatmap showed the activities of oncogenesis-related pathways in TCGA-ESCC patients, which were ordered by the expression levels of *TPM4*.
2. The ternary diagram showed the similarity/variation in RNA-seq data from control and *Tpm4* overexpressed organoids, with BS, BK and DK cells. Three independent biological replicates were performed for each group.


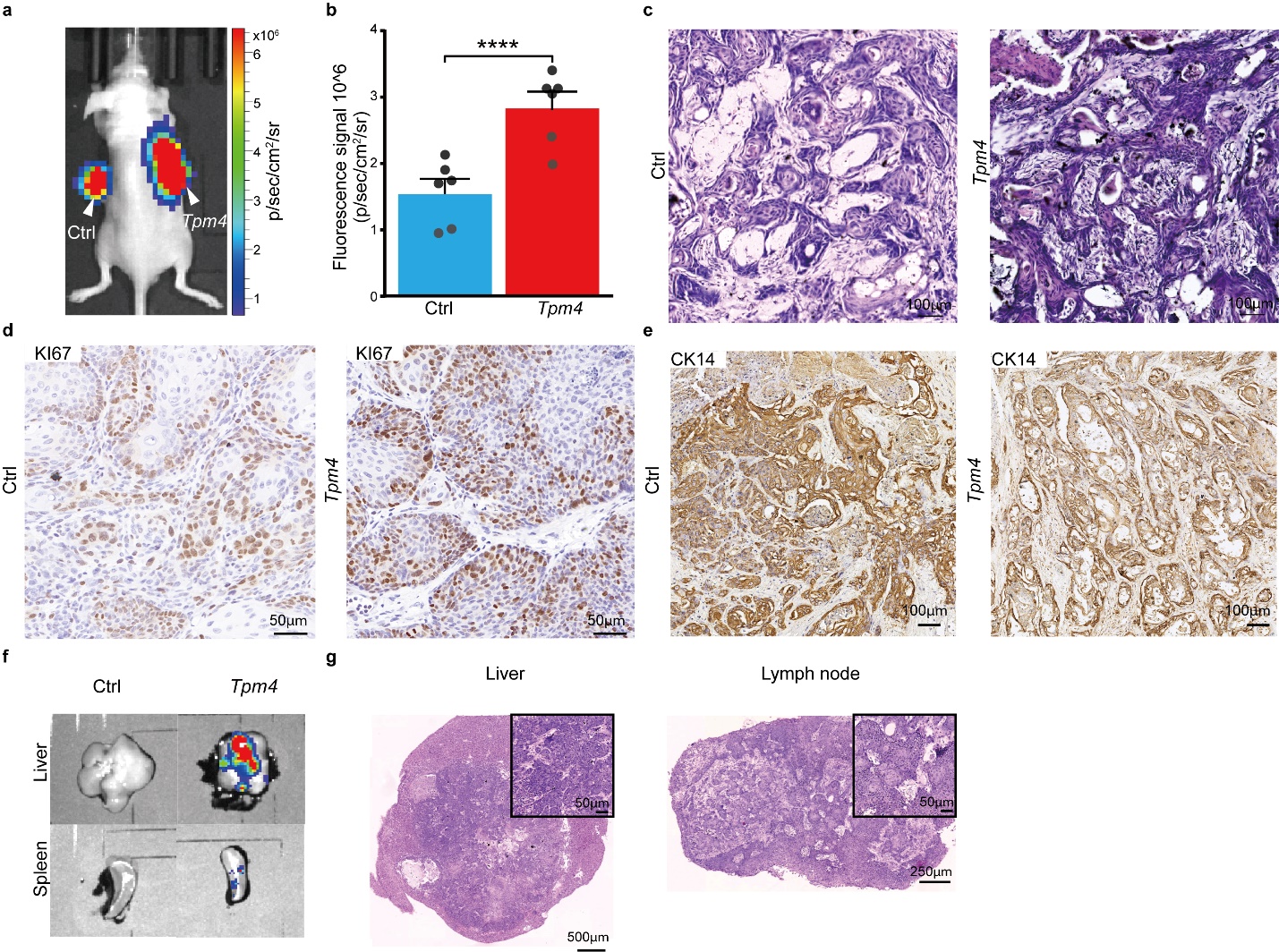


Supplementary Fig. S7. *TPM4* promotes the aggressiveness in ESCC.

1. Representative living image after subcutaneous transplanted with control (left) and *Tpm4* overexpressed tumors (right).
2. The luciferase fluorescence signal intensity of the control and *Tpm4* overexpressed mice. Data shows the means ± SEM. (n=6 biological replicates, Ctrl; n=6 biological replicates, *Tpm4*). *P* values calculated by two-sided unpaired t-test. ****, *p* < 0.0001.
3. Representative H&E staining images of subcutaneous transplantation from control (left) and *Tpm4* overexpressed (right). Scale bar, 100μm.
4. Representative immunohistochemistry images of KI67 in subcutaneous control (left) and *Tpm4* overexpressed (right) tumors. Scale bar, 50μm.
5. Representative immunohistochemistry images of CK14 in orthotopic control (left) and *Tpm4* overexpressed (right) tumors. Scale bar, 100μm.
6. The representative living image of liver (top) and spleen (bottom) in each group.
7. Representative H&E staining images of metastasis tumor from *Tpm4* overexpressed mice. The left is from the liver and the right is from the lymph node. Scale bar, 50μm, 250μm and 500μm,


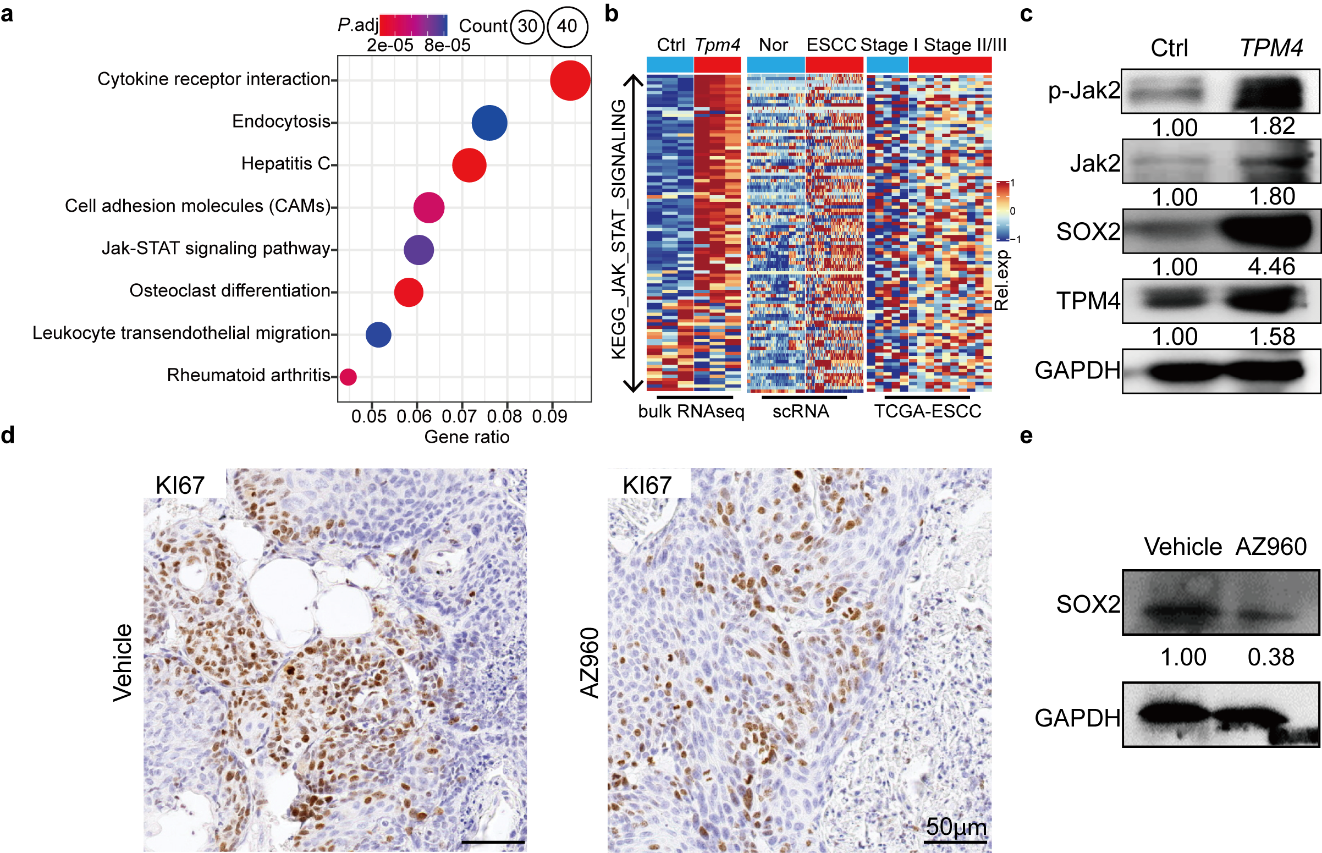


Supplementary Fig. S8. *TPM4* activates the Jak/STAT-SOX2 pathway in ESCC.

1. The KEGG enriched results in *Tpm4* overexpressed organoids compared with control.
2. The heatmap showed the expression levels of Jak/STAT pathways in RNA-seq data, scRNA-seq data and TCGA-ESCC, with the same rank order of genes.
3. The representative western blotting pictures showed the p-Jak2, Jak2, SOX2, TPM4 and GAPDH levels in the control or *TPM4* overexpressed organoids.
4. Representative IHC images of KI67 in subcutaneous *Tpm4* overexpressed tumors under vehicle (left) or AZ960 treated (right).
5. The representative western blotting pictures showed the SOX2 levels in the *Tpm4* overexpressed organoids treated with vehicle or AZ960.

Supplementary Table 1.

The top markers of subpopulation in the single-cell landscape of ESCC.

Supplementary Table 2.

The signatures of BS, BK and DK cells in single-cell normal esophageal SE cells.

Supplementary Table 3.

The ESCC aggressive stage-specific signatures were identified from GSE160269 and TCGA-ESCC.

Supplementary Table 4.

The clinical parameters in the CCI low and CCI high ESCC patiens

Supplementary Table 5.

The CCI enriched pathways.

Supplementary Table 6.

The signatures of CCI.

Supplementary Table 7.

The significantly up-regulated proteins in ESCC.

Supplementary Table 8.

The counts and differentiated expressing genes in TPM4 overexpression compared with control samples.

Supplementary Table 9

The KEGG enrichment of TPM4 overexpression compared with control samples.

Supplementary Table 10.

The counts and differentiated expressing genes in AZ960 treatment compared with vehicle groups.

Supplementary Table 11.

The sequence of guide RNA for CRISPR/cas9 knock out
